# Allele-Specific HLA Loss and Immune Escape in Lung Cancer Evolution

**DOI:** 10.1016/j.cell.2017.10.001

**Published:** 2017-11-30

**Authors:** Nicholas McGranahan, Rachel Rosenthal, Crispin T. Hiley, Andrew J. Rowan, Thomas B.K. Watkins, Gareth A. Wilson, Nicolai J. Birkbak, Selvaraju Veeriah, Peter Van Loo, Javier Herrero, Charles Swanton, the TRACERx Consortium, Charles Swanton, Mariam Jamal-Hanjani, Selvaraju Veeriah, Seema Shafi, Justyna Czyzewska-Khan, Diana Johnson, Joanne Laycock, Leticia Bosshard-Carter, Rachel Rosenthal, Pat Gorman, Robert E. Hynds, Gareth Wilson, Nicolai J. Birkbak, Thomas B.K. Watkins, Nicholas McGranahan, Stuart Horswell, Richard Mitter, Mickael Escudero, Aengus Stewart, Peter Van Loo, Andrew Rowan, Hang Xu, Samra Turajlic, Crispin Hiley, Christopher Abbosh, Jacki Goldman, Richard Kevin Stone, Tamara Denner, Nik Matthews, Greg Elgar, Sophia Ward, Marta Costa, Sharmin Begum, Ben Phillimore, Tim Chambers, Emma Nye, Sofia Graca, Maise Al Bakir, Kroopa Joshi, Andrew Furness, Assma Ben Aissa, Yien Ning Sophia Wong, Andy Georgiou, Sergio Quezada, John A. Hartley, Helen L. Lowe, Javier Herrero, David Lawrence, Martin Hayward, Nikolaos Panagiotopoulos, Shyam Kolvekar, Mary Falzon, Elaine Borg, Teresa Marafioti, Celia Simeon, Gemma Hector, Amy Smith, Marie Aranda, Marco Novelli, Dahmane Oukrif, Sam M. Janes, Ricky Thakrar, Martin Forster, Tanya Ahmad, Siow Ming Lee, Dionysis Papadatos-Pastos, Dawn Carnell, Ruheena Mendes, Jeremy George, Neal Navani, Asia Ahmed, Magali Taylor, Junaid Choudhary, Yvonne Summers, Raffaele Califano, Paul Taylor, Rajesh Shah, Piotr Krysiak, Kendadai Rammohan, Eustace Fontaine, Richard Booton, Matthew Evison, Phil Crosbie, Stuart Moss, Faiza Idries, Leena Joseph, Paul Bishop, Anshuman Chaturved, Anne Marie Quinn, Helen Doran, Angela Leek, Phil Harrison, Katrina Moore, Rachael Waddington, Juliette Novasio, Fiona Blackhall, Jane Rogan, Elaine Smith, Caroline Dive, Jonathan Tugwood, Ged Brady, Dominic G. Rothwell, Francesca Chemi, Jackie Pierce, Sakshi Gulati, Babu Naidu, Gerald Langman, Simon Trotter, Mary Bellamy, Hollie Bancroft, Amy Kerr, Salma Kadiri, Joanne Webb, Gary Middleton, Madava Djearaman, Dean Fennell, Jacqui A. Shaw, John Le Quesne, David Moore, Apostolos Nakas, Sridhar Rathinam, William Monteiro, Hilary Marshall, Louise Nelson, Jonathan Bennett, Joan Riley, Lindsay Primrose, Luke Martinson, Girija Anand, Sajid Khan, Anita Amadi, Marianne Nicolson, Keith Kerr, Shirley Palmer, Hardy Remmen, Joy Miller, Keith Buchan, Mahendran Chetty, Lesley Gomersall, Jason Lester, Alison Edwards, Fiona Morgan, Haydn Adams, Helen Davies, Malgorzata Kornaszewska, Richard Attanoos, Sara Lock, Azmina Verjee, Mairead MacKenzie, Maggie Wilcox, Harriet Bell, Allan Hackshaw, Yenting Ngai, Sean Smith, Nicole Gower, Christian Ottensmeier, Serena Chee, Benjamin Johnson, Aiman Alzetani, Emily Shaw, Eric Lim, Paulo De Sousa, Monica Tavares Barbosa, Alex Bowman, Simon Jordan, Alexandra Rice, Hilgardt Raubenheimer, Chiara Proli, Maria Elena Cufari, John Carlo Ronquillo, Angela Kwayie, Harshil Bhayani, Morag Hamilton, Yusura Bakar, Natalie Mensah, Lyn Ambrose, Anand Devaraj, Silviu Buderi, Jonathan Finch, Leire Azcarate, Hema Chavan, Sophie Green, Hillaria Mashinga, Andrew G. Nicholson, Kelvin Lau, Michael Sheaff, Peter Schmid, John Conibear, Veni Ezhil, Babikir Ismail, Melanie Irvin-sellers, Vineet Prakash, Peter Russell, Teresa Light, Tracey Horey, Sarah Danson, Jonathan Bury, John Edwards, Jennifer Hill, Sue Matthews, Yota Kitsanta, Kim Suvarna, Patricia Fisher, Allah Dino Keerio, Michael Shackcloth, John Gosney, Pieter Postmus, Sarah Feeney, Julius Asante-Siaw, Hugo J.W.L. Aerts, Stefan Dentro, Christophe Dessimoz

**Affiliations:** 1 Cancer Research UK Lung Cancer Centre of Excellence, University College London Cancer Institute, Paul O’Gorman Building, 72 Huntley Street, London WC1E 6BT, UK; 2 Division of Cancer Studies, King’s College London, Guy’s Campus, London SE1 1UL, UK; 3 Translational Cancer Therapeutics Laboratory, The Francis Crick Institute, 1 Midland Rd, London NW1 1AT, UK; 4 Cancer Genomics Laboratory, The Francis Crick Institute, 1 Midland Rd, London NW1 1AT, UK; 5 Department of Human Genetics, University of Leuven, 3000 BE Leuven, Belgium; 6 Bill Lyons Informatics Centre, University College London Cancer Institute, Paul O’Gorman Building, 72 Huntley Street, London WC1E 6BT, UK

**Keywords:** immune-escape, copy number, neoantigen, heterogeneity, cancer evolution, immune-editing, loss of heterozygosity, bioinformatics, chromosomal instability, lung cancer

## Abstract

Immune evasion is a hallmark of cancer. Losing the ability to present neoantigens through human leukocyte antigen (HLA) loss may facilitate immune evasion. However, the polymorphic nature of the locus has precluded accurate HLA copy-number analysis. Here, we present loss of heterozygosity in human leukocyte antigen (LOHHLA), a computational tool to determine HLA allele-specific copy number from sequencing data. Using LOHHLA, we find that HLA LOH occurs in 40% of non-small-cell lung cancers (NSCLCs) and is associated with a high subclonal neoantigen burden, APOBEC-mediated mutagenesis, upregulation of cytolytic activity, and PD-L1 positivity. The focal nature of HLA LOH alterations, their subclonal frequencies, enrichment in metastatic sites, and occurrence as parallel events suggests that HLA LOH is an immune escape mechanism that is subject to strong microenvironmental selection pressures later in tumor evolution. Characterizing HLA LOH with LOHHLA refines neoantigen prediction and may have implications for our understanding of resistance mechanisms and immunotherapeutic approaches targeting neoantigens.

**Video Abstract:**

## Highlights

• LOHHLA enables estimation of allele-specific HLA loss from sequencing data
• LOH of the HLA locus occurs in 40% of early stage non-small-cell lung cancers
• HLA LOH is associated with a high subclonal neoantigen burden and immune activity
• HLA LOH is an immune escape mechanism subject to strong selection pressures

## Graphical Abstract

**Figure:**
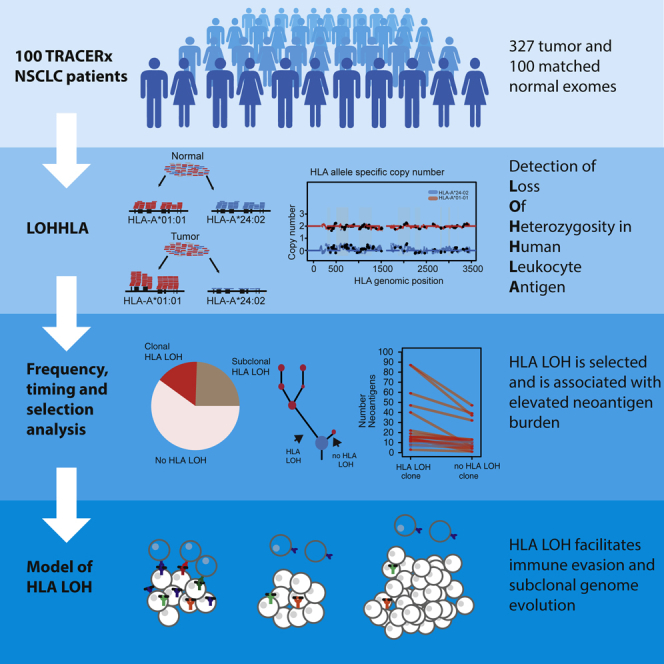

## Teaser

Development of the bioinformatics tool LOHHLA allows precise measurement of allele-specific HLA copy number, improves the accuracy in neoantigen prediction, and uncovers insights into how immune escape contributes to tumor evolution in non-small-cell lung cancer.

## Introduction

Immune evasion represents a hallmark of cancer (Hanahan and Weinberg, 2011). The majority of cancer immunotherapies, including immune checkpoint blockade therapy, aim to counteract immune evasion by shifting the balance in favor of immune activation, enabling T cell-mediated cancer cell elimination (Schumacher and Schreiber, 2015). However, only a subset of patients benefit from immunotherapies, emphasizing the need to identify the genomic and molecular determinants underpinning immune evasion.

Recent work has highlighted the importance of cancer-specific neoantigens in determining cytolytic and T cell activity as well as predicting efficacy of immune checkpoint inhibition (Brown et al., 2014, Rizvi et al., 2015, Rooney et al., 2015, Snyder et al., 2014, Van Allen et al., 2015). A critical step in neoantigen presentation and cytolytic T cell response is governed by class I human leukocyte antigen (HLA), which presents intra-cellular peptides on the cell surface for recognition by T cell receptors. Each individual’s genome contains up to six distinct HLA class I alleles, encoded by three genes (*HLA-A*, *HLA-B*, and *HLA-C*), located on the homologous paternal and maternal chromosome 6.

Downregulation of HLA genes may result in reduced antigen presentation and thus facilitate immune evasion. HLA downregulation, characterized by immunohistochemistry or monoclonal antibodies, has been found to be prevalent across a range of cancer types and has also been linked to poor outcome (Campoli and Ferrone, 2008, Hicklin et al., 1999, Hiraki et al., 2004, Mehta et al., 2008). Loss of either the maternal or paternal HLA haplotype may also impact upon the efficacy of immunotherapy. An intriguing report documented loss of heterozygosity (LOH) at the HLA locus, with loss of HLA-C^∗^08:02 in the resistant lesion from a tumor treated with tumor-infiltrating lymphocytes composed of T cell clones targeting KRAS G12D (Tran et al., 2016). Because the presence of the HLA-C^∗^08:02 allele was required for presentation of the KRAS G12D neoantigen and tumor recognition by T cells, its loss was proposed to directly enable immune evasion.

However, the impact of loss of an HLA haplotype on anti-tumor immunity, clonal expansions, and neoantigen prediction has not been systematically explored as the polymorphic nature of the HLA locus prevents alignments of sequencing reads to the human reference genome and inference of copy number. To this end, we developed LOHHLA (loss of heterozygosity in human leukocyte antigen), a computational tool permitting allele-specific copy number estimation of the HLA locus from next-generation sequencing data. Building upon previous work imputing HLA haplotypes from sequencing data (Shukla et al., 2015, Szolek et al., 2014) and utilizing previously published datasets (Brastianos et al., 2015, Jamal-Hanjani et al., 2017), we endeavored to address the prevalence and timing of HLA LOH in lung cancer and its potential impact on tumor evolution, neoantigen presentation and metastasis.

## Results

### Inferences of HLA LOH and Imbalance in Tumor Samples Using LOHHLA

In order to determine allele-specific copy number, the majority of copy-number tools rely on the relative coverage and variant allele frequency of single nucleotide polymorphisms (SNPs) in the tumor and matched normal across the genome or exome (Carter et al., 2012, Favero et al., 2015, Ha et al., 2014, Shen and Seshan, 2016, Van Loo et al., 2010). However, inferring copy number status at the HLA locus is problematic due to poor coverage and the polymorphic nature of the region. SNPs cannot readily be identified at the HLA locus using sequencing data that has been aligned to the human reference genome, as reads that are highly polymorphic will not align and will therefore be discarded. Indeed, despite being one of the most polymorphic regions of the human genome, an average of <1 (mean 0.84, range 0–7) informative heterozygous SNP in the three HLA class I genes was identified in 96 patients where copy-number analysis was possible from the TRACERx cohort (Jamal-Hanjani et al., 2017) using the state-of-the-art SNP caller Platypus (Rimmer et al., 2014). These data suggest that conventional copy-number calling algorithms are not suited to directly infer haplotype-specific copy number of the HLA locus.

**Figure 1:**
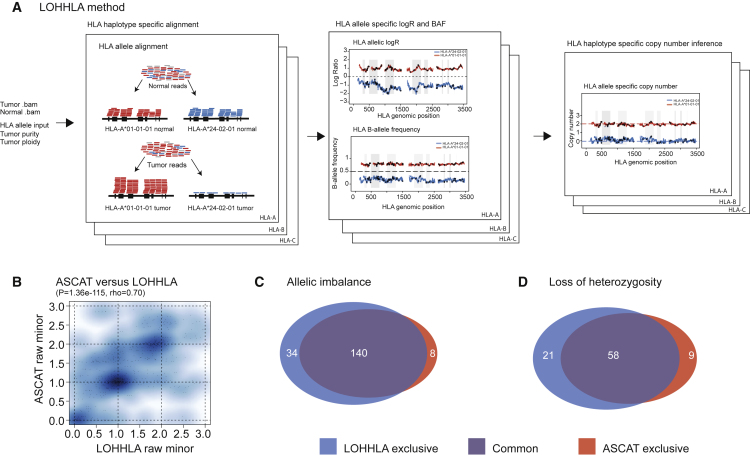
Outline and Validation of LOHHLA for Inference of HLA Class I Allele-Specific Copy Number in Tumors (A) Schematic of the LOHHLA algorithm. (B) Comparison of minor allele copy number for ASCAT and LOHHLA. (C) Venn diagram illustrating LOHHLA and ASCAT comparison for inference of allelic imbalance at HLA locus. (D) Venn diagram illustrating LOHHLA and ASCAT comparison for inference of LOH at HLA locus. See also Figures S1 and S2.

We reasoned that, by leveraging the reads that map specifically to an individual’s germline HLA alleles rather than the human reference genome, it would be possible to accurately determine HLA haplotype-specific copy number. To achieve this, we developed the computational tool LOHHLA (Figure 1A). Implementation of LOHHLA relies upon five steps. First, tumor and germline reads that map to the HLA region of the genome and chromosome 6, including contigs, are extracted. Second, tumor and germline HLA allele-specific *.bam* files are generated by aligning reads to patient-specific HLA alleles (obtained from HLA serotyping or an inference tool, e.g., Polysolver [Shukla et al., 2015] or Optitype [Szolek et al., 2014]). Third, polymorphic sites between homologous HLA alleles are identified. Fourth, tumor coverage relative to germline (logR) and b-allele frequencies (BAF) are inferred at each HLA locus, making use of identified polymorphic sites. Finally, HLA allele-specific copy number is determined for each HLA gene, accounting for stromal contamination.

**Figure S1:**
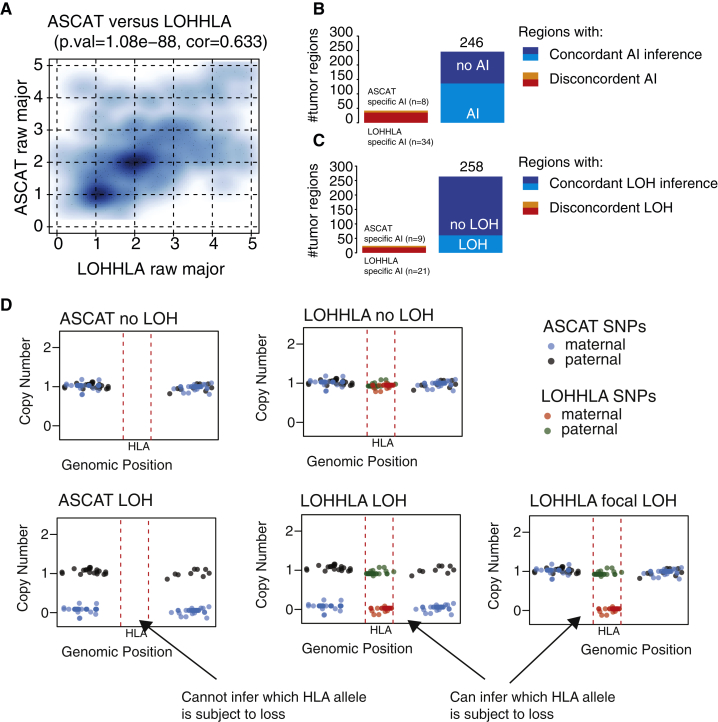
Comparison of LOHHLA and ASCAT, Related to Figure 1 (A) Plot illustrating comparison of ASCAT major copy number and LOHHLA major copy number. (B and C) Summary of concordant and discordant tumor regions in terms of allelic imbalance (B) and LOH (C). (D) Schematic illustrating how ASCAT cannot directly infer HLA copy number or which HLA allele is subject to loss. By contrast, LOHHLA uses SNPs covering HLA genes to directly infer HLA copy number.

To the best of our knowledge, no other computational method currently exists to infer haplotype-specific copy number of the HLA locus, and as such, there is no gold-standard with which we can compare LOHLA copy-number estimation or inference of which HLA haplotype is subject to loss. Therefore, to test the accuracy of HLA copy-number estimation, we made the assumption that genomic segments adjacent to the HLA locus will often exhibit the same copy-number profile as the HLA locus itself, which holds for cases without a highly focal HLA event (Figure S1D). We therefore used ASCAT (Van Loo et al., 2010) to estimate the frequency of allelic imbalance and LOH in the genomic regions surrounding the HLA locus in 288 TRACERx non-small-cell lung cancer (NSCLC) exomes from 96 patients (Jamal-Hanjani et al., 2017) and compared these to LOHHLA copy-number estimation. Notably, given that ASCAT is not designed to infer which HLA haplotype is subject to loss or imbalance, for this analysis, we could only compare whether ASCAT and LOHHLA exhibited concordant copy-number profiles not whether concordant haplotypes were predicted to be lost.

We observed a highly significant relationship between the minor and major allele copy-number estimates obtained from LOHHLA and ASCAT (p = 1.36e-115, rho = 0.70, Spearman’s rank test; Figures 1B and S1A), supporting the utility of LOHHLA to accurately estimate copy number and LOH. We found concordant allelic imbalance estimates in 246/288 tumor regions (Figures 1C, S1B, and S1C). Thirty-four additional allelic imbalance events in tumor regions were uncovered using LOHHLA while only 8 tumor regions exhibited evidence of allelic imbalance using ASCAT and not LOHHLA. In many cases, the discrepancies between ASCAT and LOHHLA could be explained by the fact that ASCAT cannot directly infer haplotype-specific copy number at the HLA locus, and thus, the copy number of either the 5′ or 3′ adjacent segment is erroneously assumed to cover the HLA locus (Figure S1D).

Concordant LOH inference, where either the maternal or paternal allele was deleted, was observed in 258/288 tumor regions, with additional LOH defined by LOHHLA identified in 21 tumor regions, while 9 tumor regions were identified as harboring a lost haplotype by ASCAT and not LOHHLA (Figures 1D and S1C).

**Figure S2:**
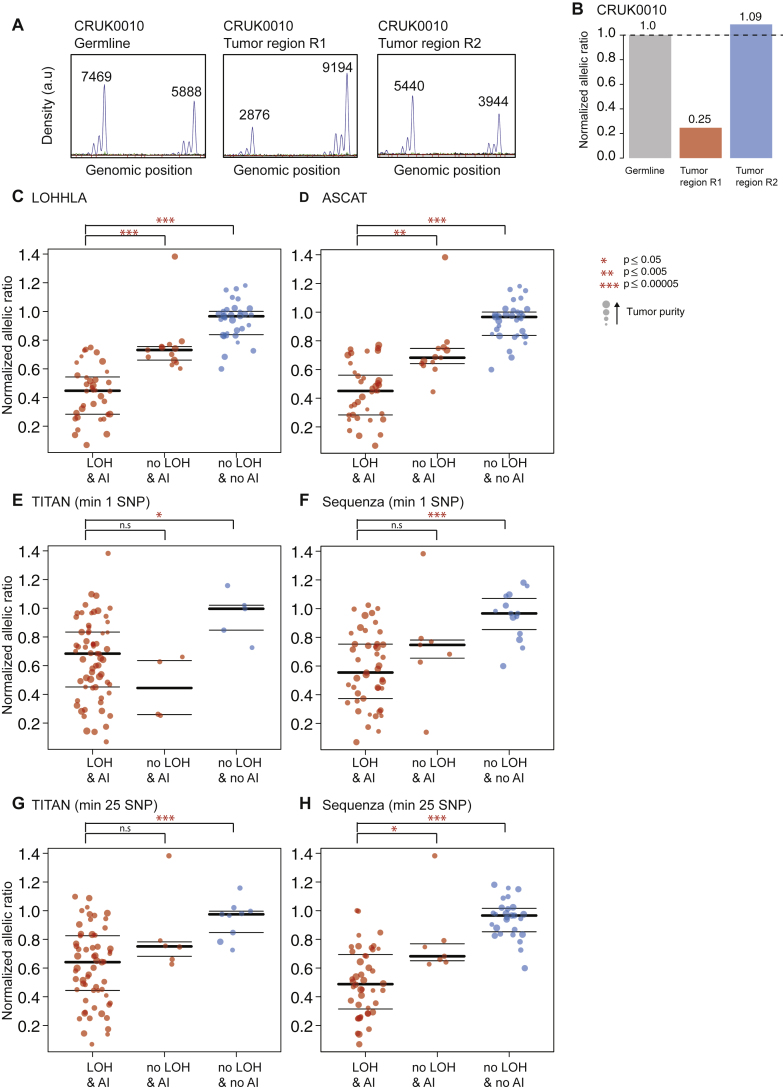
Validation of LOHHLA Using Fragment Analysis, Related to Figure 1 (A) Area under the curve of each allele using the Applied Biosystems software GeneMapper v5 for germline and tumor regions R1 and R2 in CRUK0010. (B) Normalized allelic ratio determined using the formula (A_tumor_/B_tumor_)/(A_normal_/B_normal_). Notably, region R1 shows clear evidence of allelic imbalance and likely LOH, while region R2 appears similar to germline. (C–H) Normalized allelic ratio for tumor regions showing either LOH and allelic imbalance; no LOH but allelic imbalance; or no LOH or allelic imbalance classified by LOHHLA (C), ASCAT (D), TITAN (E and G) and Sequenza (F and H). Tumor purity, as assessed by ASCAT is depicted for each tumor region, p values correspond to Wilcoxon rank sum test.

To further validate LOHHLA using an approach independent of exome sequencing, we performed PCR-based fragment analysis of highly polymorphic stretches of DNA in close proximity to the HLA locus in 82 tumor regions from 27 tumors (Figure S2). Tumor regions analyzed were either predicted to have all loci (HLA-A, HLA-B, and HLA-C) subject to LOH, or no loci affected. Supporting the utility of LOHHLA to accurately classify LOH, we observed significant differences in normalized allelic ratio between tumors classified as exhibiting LOH, allelic imbalance without LOH, or no observable imbalance (p = 1.07e-19 [LOH versus no imbalance], p = 4.57e-05 [LOH versus allelic imbalance]; Figure S2). Furthermore, the distinction between these three categories was clearer using LOHHLA than the copy-number tools ASCAT (Van Loo et al., 2010), Sequenza (Favero et al., 2015), or TITAN (Ha et al., 2014) (Figure S2).

Taken together, these data suggest that LOHHLA is able to accurately infer both allelic imbalance and LOH in tumor samples. While it may be possible to infer whether the HLA locus is subject to allelic imbalance and/or LOH in the majority of cases using copy-number tools such as ASCAT (Van Loo et al., 2010), LOHHLA provides additional sensitivity and specificity to detect these aberrations, even if they are highly focal. Crucially, LOHHLA also infers specifically which HLA allele homolog is subject to loss at each of the three HLA genes, which, to the best of our knowledge, is currently not possible with other tools.

### Prevalence and Timing of HLA Imbalance and Loss across NSCLC

**Figure 2:**
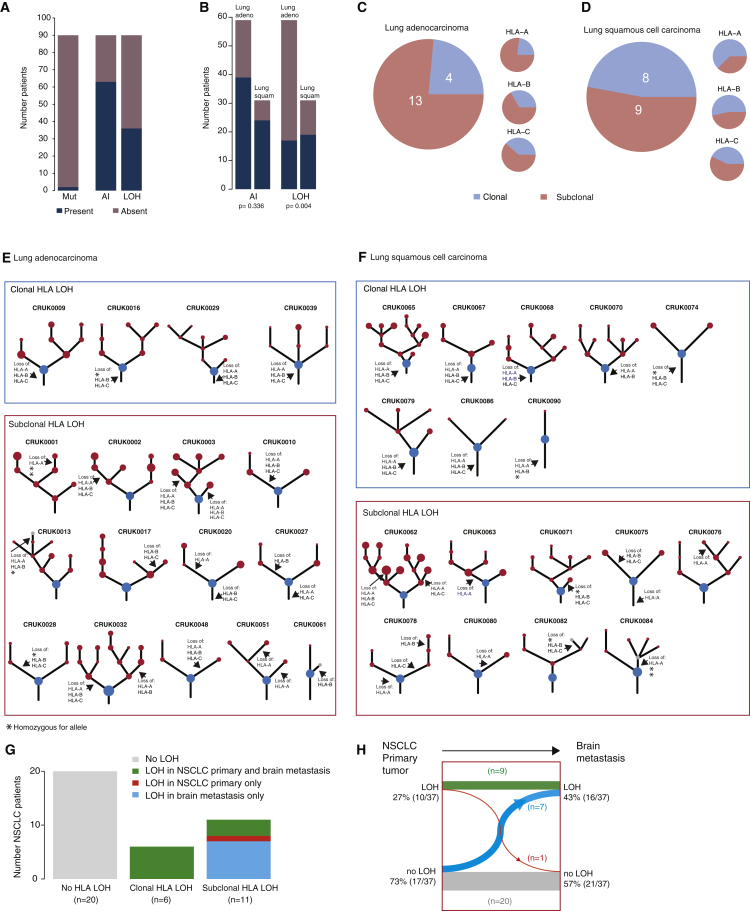
Frequency and Timing of HLA LOH in NSCLC (A) The total number of lung adenocarcinoma and lung squamous cell carcinoma TRACERx patients exhibiting an HLA non-synonymous mutation, HLA allelic imbalance (AI), or LOH at the HLA locus is shown. (B) Proportion of HLA allelic imbalance (AI) and HLA LOH identified in NSCLC by sub-type. Enrichment significance was tested using a Fisher’s exact test. (C and D) Pie charts show the timing of HLA LOH events using multi-region information for lung adenocarcinoma (C) and lung squamous cell carcinomas (D). Events at individual HLA A/B/C loci were considered clonal if they were found in every region considered and subclonal if they were found in only a subset of tumor regions. A patient sample was considered to have clonal HLA LOH if all of the individual loci lost in that tumor occurred clonally. Two lung squamous cell carcinoma patients with only a single region available for copy-number analysis are not shown. (E and F) Phylogenetic trees for each lung adenocarcinoma (E) and lung squamous cell carcinomas (F) showing evidence of HLA LOH have been annotated with the most likely timing of the HLA LOH event. Homozygous HLA alleles, where HLA LOH is not possible, are indicated by an asterisk. Clones on the phylogenetic tree (nodes) are indicated as clonal (blue) or subclonal (red). In cases where the HLA LOH event did not map to a possible clone on the phylogenetic tree, an additional gray subclone was included. (G) Number of NSCLC patients from Brastianos et al. (2015) with paired primary/brain metastasis sequencing data available exhibiting no HLA LOH (gray), HLA LOH in both the primary tumor and brain metastasis (green), HLA LOH only in the primary tumor (red), or HLA LOH only in the brain metastasis (blue). Patients with HLA LOH identified consistently across HLA loci in both the primary tumor and every brain metastases were considered to have clonal HLA LOH. Patients with inconsistent HLA loci subject to LOH or those with HLA LOH identified in only a primary or brain metastasis sample were considered to have subclonal HLA LOH. (H) Timing of the HLA LOH events. Clonal HLA LOH events occur in both the primary tumor sample and the brain metastases (green), whereas subclonal HLA LOH events either arise in the brain metastases (blue) or have occurred in a subclone of the primary tumor that does not seed the brain metastasis (red). Overall, an increase in HLA LOH is observed in the brain metastases samples as compared to the primary tumor (27% to 43%) and a corresponding decrease is observed in brain metastases samples exhibiting no HLA LOH (73% to 57%).

HLA mutations, which have the ability to disrupt neoantigen-MHC binding, have been previously described in many cancer types, including NSCLC (Shukla et al., 2015). However, despite being linked to cancer and immune escape, mutations in HLA genes are infrequently detected (Lawrence et al., 2014, Shukla et al., 2015). In our cohort of 90 lung adenocarcinoma or lung squamous cell carcinoma TRACERx patients, only tumors from three patients were found to harbor nonsynonymous mutations in HLA genes using Polysolver (Shukla et al., 2015) (Figure 2A). One lung adenocarcinoma tumor had also acquired a mutation in β-2 microglobulin (B2m), which is vital for MHC class I expression and peptide binding stability. No further mutations predicted to disrupt antigen presentation or the MHC class I complex were identified in this cohort. Likewise, a broader study of 174 lung squamous cell and 223 lung adenocarcinoma patients from TCGA only classified 8% and 5% of tumors as harboring HLA mutations, respectively (Shukla et al., 2015).

In 36/90 (40%) of NSCLCs LOHHLA identified HLA LOH, where either the maternal or paternal allele was lost, resulting in HLA homozygosity. Just as HLA mutations occur more frequently in lung squamous cell carcinomas (Shukla et al., 2015), we also observed an enrichment for HLA LOH in this histological subtype (p = 0.004, 19/31 [61%] of lung squamous cell carcinomas versus 17/59 [29%] of lung adenocarcinomas) (Figures 2A and 2B). The high frequency with which HLA LOH occurs and the possibility of previously antigenic peptides no longer being presented on the lost allele suggests that HLA LOH has the capacity to be a more prevalent mechanism of immune disruption than HLA or B2M mutations.

To investigate whether HLA allele-specific loss was an early event in the tumor’s evolution, present clonally in every cancer cell, or whether it was present subclonally, in only a subset of cancer cells, indicating an occurrence later in evolution and potentially in response to a shift in the equilibrium between immune recognition and evasion, we utilized the high-depth and multi-region nature of the TRACERx dataset. In this cohort of early stage NSCLC tumors, HLA LOH appeared to frequently occur subclonally in both histological subtypes, with 13/17 lung adenocarcinoma and 9/17 lung squamous cell carcinomas exhibiting loss of an HLA allele in a subset of cancer cells (Figures 2C and 2D). Clonality of the HLA LOH event could not be determined for two lung squamous cell carcinoma patients with only a single region available for copy-number analysis. Phylogenetic analysis permitted us to map HLA LOH events to probable subclones from the tumor’s evolutionary tree (Figures 2E and 2F) (Jamal-Hanjani et al., 2017). These data suggest that selective pressure from the immune system may increase during tumor development and also that without multi-region sequencing, the prevalence of HLA LOH may be significantly underestimated.

To shed further light on the timing of HLA LOH in NSCLC tumor evolution, we obtained sequencing data for 37 NSCLC primary tumors with matched brain metastases (Brastianos et al., 2015). Consistent with data from early stage NSCLC, we identified HLA LOH in 17/37 (46%) tumors and found that the LOH event occurred subclonally in 11/17 (65%) cases in which it occurred (Figure 2G). Furthermore, when we compared primary and metastatic samples taken from the same patient, we observed a trend toward enrichment of HLA LOH in brain metastases compared to the matched primary tumor (p = 0.08, McNemar’s test), with seven patients harboring HLA LOH in the metastatic sample alone and only one patient where the converse was observed, with HLA LOH in the primary tumor alone (Figure 2H). These results support the notion of HLA LOH occurring later in cancer evolution and indicate that there may be selection for immune evasive mechanisms in late stage disease.

### HLA Loss Is under Positive Selection in NSCLC

**Figure 3:**
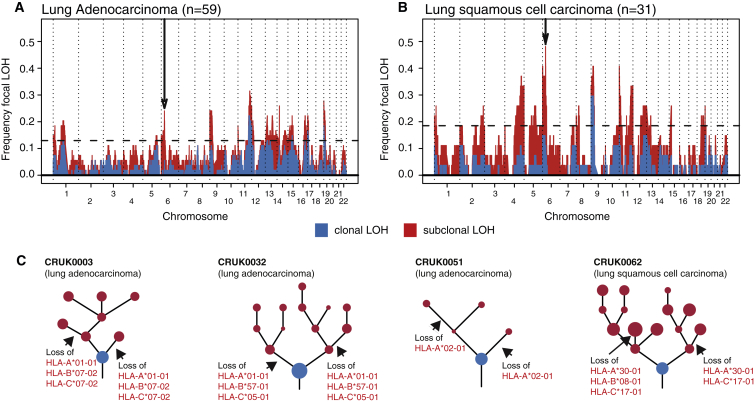
HLA LOH Reflects Selection in NSCLC (A and B) Frequency of focal LOH in lung adenocarcinoma (A) and lung squamous cell carcinoma (B). Focal LOH is defined as <75% of a chromosome arm. Arrow indicates location of HLA locus. Horizontal dashed line depicts significant focal LOH at p = 0.05, using simulations. Clonal LOH is shown in blue, with subclonal LOH shown in red. Chromosome arm LOH and focal subclonal LOH is shown in Figure S3. (C) Parallel evolution of HLA LOH, with allele-specific HLA loss shown on phylogenetic trees. See also Figure S3.

**Figure S3:**
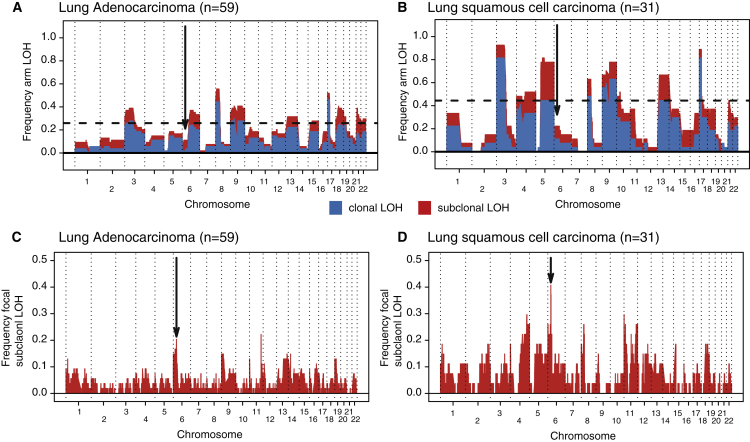
Arm-Level and Focal Subclonal LOH across the Genome, Related to Figure 3 (A and B) Arm-level LOH across the genome for lung adenocarcinoma (A) and lung squamous cell carcinoma (B). Arm-level LOH is defined as > 75% of a chromosome arm. Arrow indicates location of HLA locus. Horizontal dashed line depicts significant focal LOH at p = 0.05, using simulations. Clonal LOH is shown in blue, with subclonal LOH shown in red. (C and D) Focal subclonal LOH across the genome for lung adenocarcinoma (C) and lung squamous cell carcinoma (D). Focal LOH is defined as < 75% of a chromosome arm. Arrow indicates location of HLA locus.

Given the relevance to immune evasion and high incidence of both clonal and subclonal LOH in HLA genes, we asked whether HLA LOH was significantly more frequent than expected by chance. Taking the frequency of LOH in every tumor into account, we simulated the expected frequencies of both focal and arm-level events. The observed incidence of focal, but not arm-level, HLA LOH occurred at a significantly greater frequency than expected by chance (Figures 3, p < 0.001, and S3). Indeed, we observed a clear peak in focal LOH centered around the HLA locus for both histological subtypes. This peak was more pronounced when restricting the analysis to subclonal LOH (Figure S3). Thus, while chromosomal instability may lead to LOH at the HLA locus, facilitating immune escape, the high prevalence of HLA LOH, beyond that expected by chance, suggests it is subject to significant positive selection in tumor evolution.

Moreover, in keeping with a strong selective pressure later in tumor evolution, in four tumors we observed losses of HLA haplotypes occurring as distinct events on separate branches of the tumors’ phylogenetic trees, indicative of parallel evolution with convergence upon HLA loss (Figure 3C). Of note, in all four cases where we observed parallel evolution, the same alleles were subject to loss on distinct branches, suggesting that loss of these alleles specifically may have been required for subclonal expansions. We also noted that in certain cases (e.g., CRUK0051) only one HLA gene was subject to allele-specific loss, implying a selective benefit of perturbations to neoantigen presentation associated with that gene specifically.

Taken together with the recently described significant mutation frequency in HLA genes across tumors (Lawrence et al., 2014, Shukla et al., 2015), these data implicate HLA LOH as a common mechanism of immune evasion in lung cancer evolution. Furthermore, these data suggest that the immune system acts as a strong selection pressure during branched tumor development.

It is also notable that while HLA LOH was identified in 36 tumors, we did not identify any tumors exhibiting homozygous deletions of HLA. Concordant with this observation, the variant allele frequencies of mutations that have been identified in HLA genes are indicative of a heterozygous state (Shukla et al., 2015). These data support the notion that a single copy of an HLA haplotype may be mandatory to avoid NK-mediated target cell lysis (Moretta et al., 2014).

### HLA Loss Reflects Immune Editing and Is Associated with an Enrichment of Subclonal Mutations

Conceivably, if one of the homologous chromosomes harboring the HLA haplotypes were subject to copy-number loss, the number of putative neoantigens presented to T cells would be reduced. Thus, we hypothesized that loss of an HLA haplotype may be permissive for subclonal expansions and would be associated with an elevated mutation/neoantigen burden.

**Figure 4:**
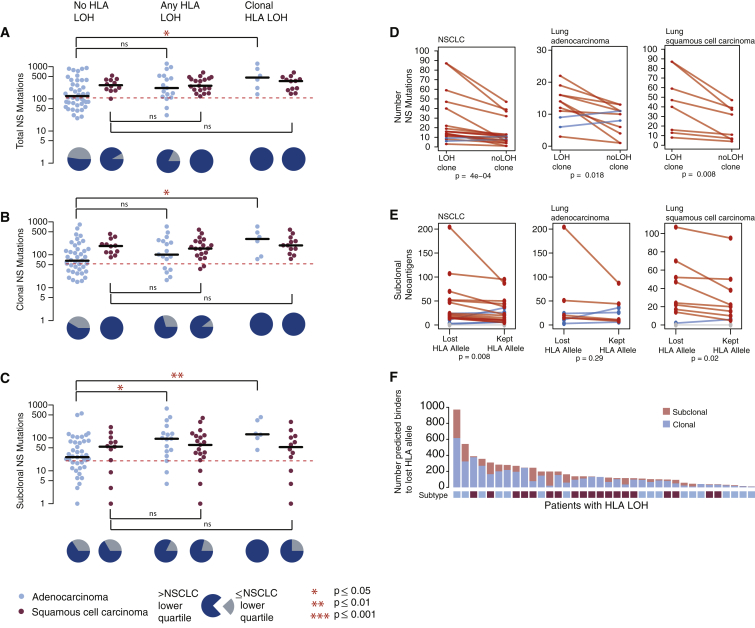
Non-synonymous Mutational Burden Associates with HLA LOH, and Neoantigens More Frequently Bind the Lost Allele (A) The total number of nonsynonymous mutations is plotted across different categories of HLA LOH for lung adenocarcinoma (light blue) and lung squamous cell carcinomas (magenta). Tumors were classified as having: no HLA LOH; any HLA LOH event, without taking into account the timing of the event; or clonal HLA LOH. The lowest total non-synonymous mutation quartile is indicated by the dashed red line and the proportion of tumors with a total non-synonymous mutational burden greater or less than that is indicated by the pie charts for each HLA LOH classification group. (B) The number of clonal non-synonymous mutations is plotted across different categories of HLA LOH for lung adenocarcinoma (light blue) and lung squamous cell carcinomas (magenta). (C) The number of subclonal non-synonymous mutations is plotted across different categories of HLA LOH for lung adenocarcinoma (light blue) and lung squamous cell carcinomas (magenta). All p values are calculated using an unpaired Wilcoxon test. (D) The number of non-synonymous mutations found in the clone harboring the HLA LOH event compared to the number of non-synonymous mutations in its sister clone, descended from the same ancestral cancer cell, but without HLA LOH. The p value is calculated using a paired Wilcoxon test. (E) The number of subclonal neoantigens predicted to bind to either the lost HLA allele or the kept HLA allele is indicated for all NSCLC tumors exhibiting HLA LOH, all lung adenocarcinoma tumors with HLA LOH, and all lung squamous tumors with HLA LOH. A red line indicates an elevated subclonal neoantigen mutation burden in the HLA LOH subclone compared to the subclone without HLA LOH, while blue indicates the converse. The p value is calculated using a paired Wilcoxon test. (F) The total number of mutations predicted to result in a binder to the lost allele is shown for all patients with at least one HLA LOH event. The mutation clonality is also indicated as either clonal (light blue) or subclonal (light red). See also Figure S4.

**Figure S4:**
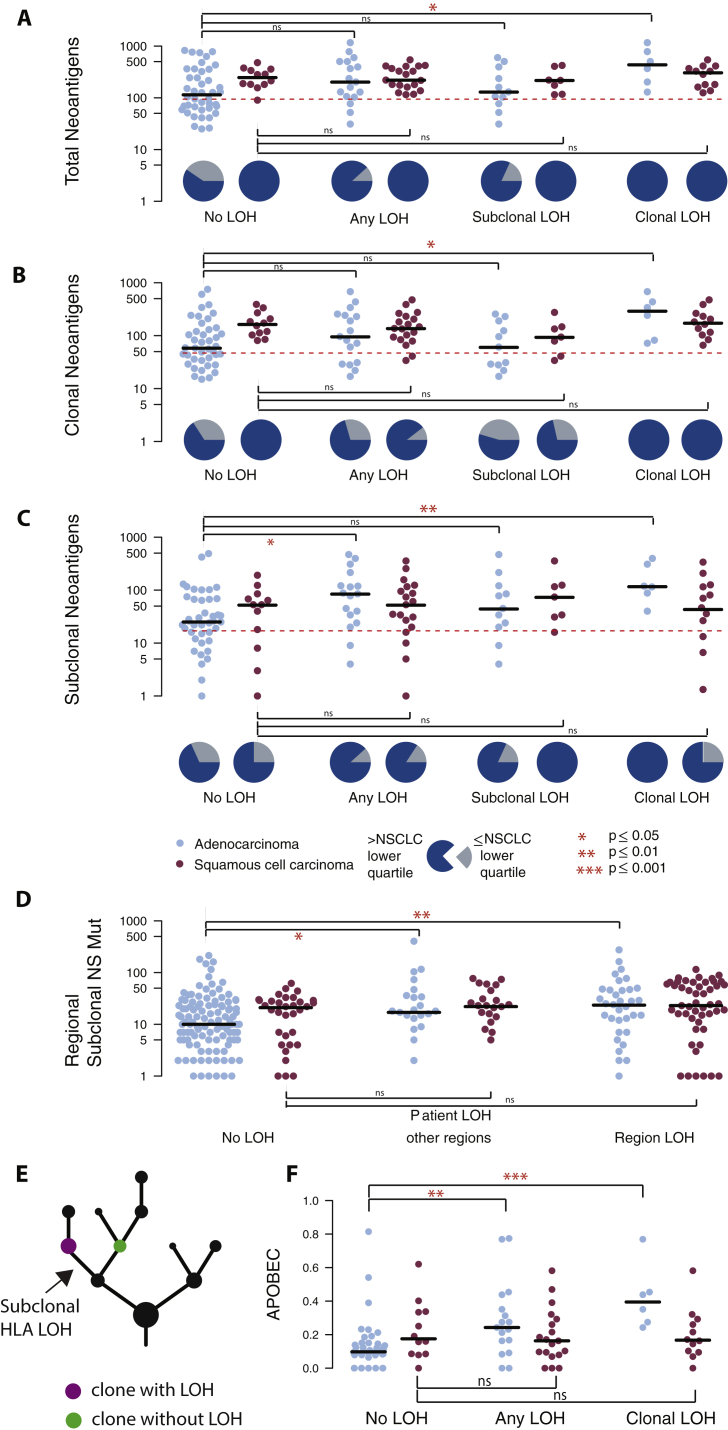
Neoantigen and Regional HLA LOH Associations, Related to Figure 4 (A) The total number of neoantigens is plotted across different categories of HLA LOH for lung adenocarcinoma (light blue) and lung squamous cell carcinomas (magenta). Tumors were classified as having: no HLA LOH; any HLA LOH event, without taking into account the timing of the event; subclonal HLA LOH; or clonal HLA LOH. The lowest total neoantigen quartile is indicated by the dashed red line and the proportion of tumors with a total neoantigen burden greater or less than that is indicated by the pie charts for each HLA LOH classification group. (B) The number of clonal neoantigens is plotted across different categories of HLA LOH for lung adenocarcinoma (light blue) and lung squamous cell carcinomas (magenta). (C) The number of subclonal neoantigens is plotted across different categories of HLA LOH for lung adenocarcinoma (light blue) and lung squamous cell carcinomas (magenta). (D) The number of subclonal non-synonymous mutations is plotted for tumor regions from tumors without any indication of HLA LOH, for tumor regions without HLA LOH from a tumor with other regions harboring HLA LOH, and for tumor regions containing an HLA LOH event. All p values are calculated using an unpaired wilcoxon test. (E) Schematic of the clones considered for the comparison performed in Figure 4D. Here, the cancer subclone harboring HLA loss (purple) is shown with its sister subclone, descended from the same ancestral cancer cell, but without HLA loss (green). (F) For each lung adenocarcinoma (blue) and lung squamous cell carcinoma (purple) tumor, the relative contributions of APOBEC mutational signatures are shown. p values are calculated using an unpaired wilcoxon test.

We first compared the number of non-synonymous mutations and neoantigens present in tumor samples with and without LOH at the HLA locus, without taking into account timing or clonal nature of the HLA LOH event. While overall, we observed a significant increase in the number of non-synonymous mutations (Figure 4A) and neoantigens (Figure S4A) in tumor samples exhibiting any HLA LOH, this did not remain significant when the subtypes were considered separately (NSCLC p = 0.016; lung adenocarcinoma p = 0.07; lung squamous cell carcinoma p = 0.82, Wilcoxon test). However, we observed only 3/36 tumors with HLA LOH that exhibited a low mutational burden (as defined by the lowest quartile of NSCLC mutation burden), compared to 21/54 tumors without HLA LOH.

When we considered the clonal nature of mutations, we found that among tumors with HLA LOH there was a significant increase in the number of subclonal, but not clonal, non-synonymous mutations (Figures 4B and 4C) (NSCLC p = 0.008; lung adenocarcinoma p = 0.01; lung squamous cell carcinoma p = 0.6, Wilcoxon test) and neoantigens (Figures S4B and S4C). This observation is consistent with HLA LOH frequently occurring as a branched, subclonal event and indicates that HLA LOH may allow for the accumulation of potentially antigenic subclonal mutations. Consistent with this, we found that when HLA LOH occurred as a clonal event, on the trunk of a tumor’s phylogenetic tree, this was significantly associated with both an elevated clonal (NSCLC p = 0.002; lung adenocarcinoma p = 0.01; lung squamous cell carcinoma p = 0.29, Wilcoxon test) and subclonal (NSCLC p = 0.03; lung adenocarcinoma p = 0.004; lung squamous cell carcinoma p = 0.89, Wilcoxon test) non-synonymous mutation and neoantigen burden (Figures 4B, 4C, S4B, and S4C).

When we considered HLA LOH events at the region-level, we also observed a significant increase in subclonal mutations between tumor regions exhibiting HLA loss compared to tumor regions from patients without any evidence for HLA LOH (Figure S4D; NSCLC p = 1.9e-05; lung adenocarcinoma p = 0.009; lung squamous cell carcinoma p = 0.07). Interestingly, even in tumor regions without HLA LOH, but evidence for HLA LOH in other regions from the same tumor, we observed a significantly higher burden of subclonal mutations compared to tumor regions derived from tumors without any evidence for HLA LOH (Figure S4D). Thus, while HLA LOH may allow for subsequent subclonal expansion, a tumor with a high mutational burden may be under increased selective pressure for the HLA LOH event.

We next considered the specific cancer subclones in which HLA LOH events occurred, allowing us to more directly assess the impact of HLA LOH on non-synonymous mutation and neoantigen burden in cancer cells (Figure S4E). In tumors with subclonal HLA LOH, we directly compared the mutational burden of the cancer subclone harboring HLA loss with its sister subclone, descended from the same ancestral cancer cell, but without HLA loss. Among the 36 tumors exhibiting any HLA LOH, we identified 19 instances where the event was subclonal and not on a terminal node for which a comparison between sister subclones could be made. Subclones with HLA LOH consistently showed a higher non-synonymous mutational burden than their counterparts without HLA LOH, regardless of histological subtype (Figure 4D; NSCLC p = 4e-04; lung adenocarcinoma p = 0.018; lung squamous cell carcinoma p = 0.008). Indeed, there were only 2/19 instances of the subclone with HLA LOH having fewer non-synonymous mutations than its sister subclone without HLA LOH. This result suggests that HLA LOH may contribute to the observed increase in subclonal non-synonymous mutations among tumors harboring HLA LOH.

While there were only three instances of low mutational burden in tumors harboring an HLA LOH event (Figure 4A) and an increase in mutation burden in subclones harboring HLA LOH was observed in both cancer types, we noted that a significant increase in subclonal non-synonymous mutation burden in tumors with loss of an HLA allele compared to those without HLA LOH was only observed among the lung adenocarcinomas. These data suggest that while HLA LOH may allow for acquisition of subclonal mutations in lung squamous cell carcinomas, there are likely to be additional mechanisms contributing to the observed high subclonal mutational burden in tumors without HLA LOH in this subtype.

To address whether a particular mutational process contributes to the subclonal mutational burden present in tumors with HLA LOH, we interrogated the mutational signatures present in each tumor (Alexandrov et al., 2013, Rosenthal et al., 2016). Among lung adenocarcinoma tumors that exhibited any HLA LOH, we observed a significant increase in the APOBEC mutagenic signatures (Signature 2 and Signature 13) (NSCLC p = 0.03; lung adenocarcinoma p = 0.003, lung squamous cell carcinoma p = 0.63, Figure S4F); however, no other signature found in this cohort (Signatures 1A, 4, and 5) appeared to differentially contribute between groups.

Only neoantigens binding to the kept HLA alleles will be presented to the immune system. We reasoned that if HLA LOH reflects cancer immune-editing one would expect to observe an enrichment of subclonal neoantigens predicted to bind with high affinity to the lost HLA alleles compared to the kept HLA alleles. We therefore investigated tumors with six distinct HLA alleles and loss of one HLA haplotype (HLA-A, HLA-B, and HLA-C) in at least one tumor region (n = 20; 9 lung adenocarcinomas and 11 lung squamous cell carcinoma). Consistent with LOH at the HLA locus representing immune editing and facilitating accumulation of subclonal neoantigens, we observed a significant enrichment for subclonal neoantigens predicted to bind to the lost HLA alleles compared to the kept alleles (Figure 4E) (NSCLC p = 0.0083; lung adenocarcinoma p = 0.29; lung squamous cell carcinoma p = 0.02, paired Wilcoxon test). In one extreme example, tumor CRUK0020, a lung adenocarcinoma, we observed a total of 1,220 mutations predicted to yield neoantigens, of which 92% were predicted to bind to lost HLA alleles.

To determine more generally the impact HLA LOH might have on which neoantigens are presented to the immune system, we identified neoantigens predicted to bind to lost alleles in the full cohort of 36 patients exhibiting any HLA LOH (Figure 4F). We found that all patients harbored mutations predicted to bind to a now lost HLA allele, highlighting the potential impact HLA LOH could have on the targeting of putative neoantigens in a clinical setting, such as through personalized neoantigen vaccine approaches (Ott et al., 2017, Sahin et al., 2017).

### HLA Loss and Immune Phenotype

Next, to investigate whether HLA loss might be associated with an immune replete tumor microenvironment, we performed immunohistochemistry analysis to determine the expression of PD-L1 on both tumor and immune cells. PD-L1 is a ligand to the immune inhibitory receptor PD1 and its expression may reflect a cancer adaptive immune response to an active immune system.

**Figure 5:**
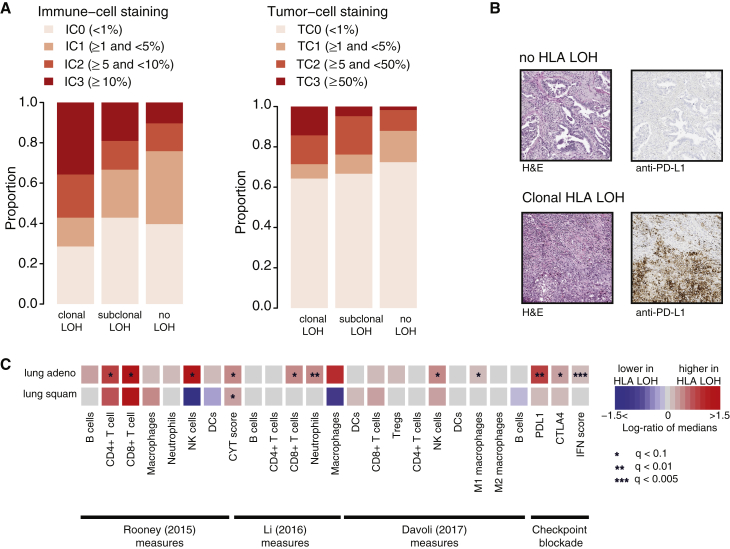
HLA LOH and Immune Phenotypes (A) Anti-PD-L1 staining on FFPE diagnostic blocks from tumors with clonal HLA LOH, subclonal HLA LOH, and no observed HLA LOH. Immune-cell-based staining and tumor-cell staining is depicted. (B) Staining from two representative tumors, one without HLA LOH and one with clonal HLA LOH is shown. (C) The log-ratio of medians between tumors containing an HLA LOH event at all loci and those without any HLA LOH event is shown for published immune microenvironment measures and signatures. Increase of an immune measure among tumors with HLA LOH is shown in red, and a decrease is shown in blue. False discovery rate (FDR) (q) values comparing the distribution of immune measures between the HLA LOH groups are indicated by asterisks (^∗^). See also Figure S5 and Table S1.

We found tumors exhibiting clonal HLA LOH were characterized by significantly elevated PD-L1 staining of immune cells compared to tumors without any HLA LOH (p = 0.029, Cochrane Armitage test), and a trend was observed for elevated PD-L1 staining on tumor cells (p = 0.14, Cochrane Armitage test). These data are consistent with the notion that HLA LOH may facilitate immune escape in response to an active immune microenvironment (Figures 5A and 5B).

To further validate our findings in a larger cohort with RNA-seq data, we obtained 383 lung adenocarcinomas and 309 lung squamous-cell carcinomas samples from TCGA (Campbell et al., 2016).

**Figure S5:**
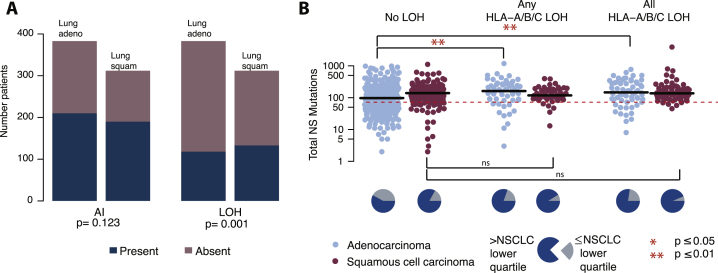
Frequency and Association with Mutational Burden of HLA LOH in TCGA, Related to Figure 5 (A) The total number of TCGA patients exhibiting an allelic imbalance or LOH at the HLA locus is shown. (B) The total number of nonsynonymous mutations is plotted across different categories of HLA LOH for lung adenocarcinoma (light blue) and lung squamous cell carcinomas (magenta). Tumors were classified as having: no HLA LOH; any HLA LOH event; or HLA LOH at all three HLA loci. The lowest total non-synonymous mutation quartile is indicated by the dashed red line and the proportion of tumors with a total non-synonymous mutational burden greater or less than that is indicated by the pie charts for each HLA LOH classification group.

In keeping with results from the TRACERx cohort, we found HLA LOH was highly prevalent in lung squamous-cell carcinomas (133/309) and lung adenocarcinomas (118/383) tumors and significantly enriched in lung squamous cell carcinomas compared to adenocarcinomas (p = 0.001, Fisher’s exact test) (Figure S5A). Additionally, we again observed a significantly higher non-synonymous mutation burden in lung adenocarcinomas tumors exhibiting HLA LOH (p = 0.0001, Wilcoxon test), regardless of whether the HLA LOH affected a single locus (p = 0.002, Wilcoxon test) or all three HLA loci (p = 0.003, Wilcoxon test) (Figure S5B), a factor we could now consider due to the increased sample size from TCGA.

Previous work has identified immune signatures indicative of immune activity and/or immune cell infiltrates (Davoli et al., 2017, Li et al., 2016, Rooney et al., 2015). By using these signatures, we were able to further investigate whether HLA loss was associated with a specific immune phenotype. Consistent with the immunohistochemistry results, in both lung adenocarcinoma and lung squamous cell carcinomas harboring HLA LOH, we identified a significantly elevated cytolytic activity score, which measures the levels of two genes upregulated upon CD8^+^ T cell activation, granzyme A (GZMA) and perforin (PRF1) (Rooney et al., 2015) (Figure 5C). In lung adenocarcinoma with HLA LOH at all three loci, we observed an increase in abundance of CD8^+^ T cells and expression profiles associated with improved checkpoint blockade response (Herbst et al., 2014, Li et al., 2016, Piha-Paul et al., 2016, Ribas et al., 2015, Rooney et al., 2015, Tumeh et al., 2014). Additionally, we identified an increase in NK cells, suggesting that HLA LOH alone may interrupt inhibitory NK cell/MHC interactions (Figure 5C). Differential expression analysis between tumors with and without LOH confirmed an increase of PD-L1 and effector molecules such as granzymes-A, -B, and -H, as well as STAT1 and interferon (IFN)-γ, in lung adenocarcinoma with HLA LOH but not lung squamous cell carcinoma (Table S1).

These data suggest that lung tumors with HLA loss have a more active immune predatory microenvironment and disruption of antigen presentation may act as a mechanism to evade the immune system.

## Discussion

**Figure 6:**
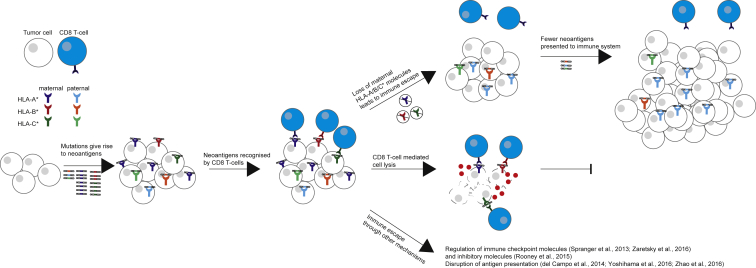
Model of HLA Allele-Specific Loss in NSCLC Model illustrating how HLA LOH may lead to immune escape in tumors. During tumor evolution, the accumulation of neoantigens may induce local immune infiltrates, including CD8 T cells. Local immune infiltrates may act as a selection barrier for tumors. Subclones with HLA LOH may be positively selected as these can evade killing by avoiding CD8 T cell recognition. Alternatively, other subclones may evade killing through other mechanisms.

Losing the ability to present productive tumor neoantigens could facilitate evasion from immune predation. An integral part of neoantigen presentation is the HLA class I molecule, which presents epitopes to T cells on the cell surface. Thus, loss of an HLA allele, resulting in HLA homozygosity, may be a mechanism of immune escape (Figure 6).

However, the polymorphic nature of the HLA locus precludes accurate copy-number calling using conventional copy-number tools. Here, we present LOHHLA, a computational tool to systematically evaluate the prevalence and importance of HLA loss in lung cancer evolution using next-generation sequencing data (Figure 1).

We evaluated the performance of LOHHLA using two independent methods. We found LOHHLA LOH and allelic imbalance estimates were consistently in agreement with those inferred from adjacent genomic segments using the state-of-the-art copy-number tool ASCAT (Van Loo et al., 2010). PCR-based fragment analyses of polymorphic stretches of DNA validated the accuracy of LOHHLA using an approach independent of exome sequencing. Importantly, LOHHLA is able to determine which specific HLA haplotype is subject to copy-number loss, which is not possible using conventional copy-number tools.

Using LOHHLA, we find that HLA loss occurs in 40% of early-stage NSCLCs. The focal nature and high frequency, beyond that expected using simulations, suggest HLA LOH is strongly selected for in NSCLC evolution. The subclonal frequency of HLA loss, occurring in a subset of cancer cells, on the branches of the tumors’ phylogenetic trees, suggests it is often a later event in tumor evolution and that the local, region-specific, immune microenvironment may act as a key selective force in shaping branched tumor evolution. In keeping with these results, in four early stage tumors, we observed evidence for parallel evolution of HLA allele-specific loss, and in a cohort of primary NSCLC tumors with matched brain metastasis (Brastianos et al., 2015), we detected HLA LOH in 47% of cases, occurring subclonally in the majority of cases (11/17) and preferentially at the metastatic sites (Figure 3H). These results support the notion that escape from immune predation represents a significant constraint to tumor evolution. These observations have parallels with HIV evolution whereby patients with homozygous HLA alleles exhibit more rapid progression to AIDS compared to patients with heterozygous HLA alleles (Martin and Carrington, 2013).

In both lung adenocarcinomas and lung squamous cell carcinomas, subclones harboring HLA LOH were associated with a significantly elevated non-synonymous mutation/neoantigen burden compared to subclones descended from the same ancestral cancer cell but without HLA LOH. Tumors with HLA LOH were found to exhibit an enrichment of neoantigens predicted to bind to the lost HLA alleles and were associated with significantly elevated PD-L1 staining on immune cells and RNA signatures of immune activation. These data suggest that loss of HLA alleles, under the selective pressure of immune predation, may be permissive for subclonal expansions and result in previously antigenic mutations becoming effectively invisible to the immune system.

The high mutational load and low levels of HLA expression in lung squamous cell tumors (McGranahan et al., 2016), even in tumors without HLA LOH suggests alternative mechanisms of immune evasion and/or disruption of neoantigen presentation through other mechanisms (e.g., mutations to *B2M* or *NLRC5*) (del Campo et al., 2014, Yoshihama et al., 2016). In this regard, we note that LOHHLA could be extended to perform haplotype-specific copy number on any genomic segment that has been subject to haplotyping. For instance, if HLA class II typing has been performed, LOHHLA can be implemented to assess the extent to which loss of HLA class II occurs in tumor evolution and which haplotype is subject to loss.

Further work is warranted to explore the extent to which HLA LOH represents a pan-cancer immune evasion mechanism. Immunohistochemistry analysis has documented loss of HLA expression in many cancers (Campoli and Ferrone, 2008, Hicklin et al., 1999, Mehta et al., 2008), however, the extent to which allele-specific loss of HLA molecules is a pervasive mechanism of immune evasion in tumor evolution across cancer types remains unclear. Furthermore, as more data pre- and post-therapy emerges, it will be possible to investigate the extent to which HLA LOH represents a common mechanism of resistance within the context of checkpoint blockade (and other immune-targeted) therapies.

Our results may also have implications for vaccine- and T cell-based therapeutic approaches, specifically targeting neoantigens, with up to 92% predicted neoantigens in one tumor found to bind the lost haplotype. Indeed, consistent with the findings of Tran et al. (2016), these findings support the notion that taking into account HLA LOH might help determine which set of predicted neoantigens are more likely to elicit an effective T cell response.

In conclusion, LOHHLA enables accurate estimation of haplotype-specific HLA loss from sequencing data, revealing that HLA LOH is a common feature of NSCLC, facilitating immune escape and subclonal genome evolution.

## STAR★Methods

### Key Resources Table

**Table:** 

REAGENT or RESOURCE	SOURCE	IDENTIFIER
**Biological Samples**		

TRACERx 100	Jamal-Hanjani et al., 2017	N/A

**Oligonucleotides**		

Primer, D6S2852: Forward: TTCAGTGAATCATGAGCATG	https://genome.ucsc.edu	D6S2852_ FAM_F
Primer, D6S2852: Reverse: TGCAAGTGCTCAATGCAGCC	https://genome.ucsc.edu	D6S2852_R
Primer, D6S2872: Forward: CACAGCAGGAAAGGGTTGAC	https://genome.ucsc.edu	D6S2872_HEX_F
Primer, D6S2872: Reverse: CCATGAAAAAGTCTGTCCCG	https://genome.ucsc.edu	D6S2872_R
Primer, D6S248: Forward: TTGCAGTGAGCCGAGATCAA	https://genome.ucsc.edu	D6S248_FAM_F
Primer, D6S248: Reverse: GACAATATCAAAAAGAACTGCCAAA	https://genome.ucsc.edu	D6S248_R
Primer, D6S1022: Forward: AAAGTGAGACTCCGCCTCAT	https://genome.ucsc.edu	D6S1022_HEX_F
Primer, D6S1022: Reverse: CACCTCAGCCTCTTTGGTAG	https://genome.ucsc.edu	D6S1022_R

**Antibodies**		

Anti-human PD-L1 rabbit monoclonal antibody	Ventana, Tucson, AZ	SP142

**Deposited Data**		

TRACERx raw and analyzed data	Jamal-Hanjani et al., 2017	EGAS00001002247
TCGA NSCLC data	Campbell et al., 2016	https://gdc.cancer.gov

**Software and Algorithms**		

Samtools	Li and Durbin, 2009	http://samtools.sourceforge.net/
GATK	McKenna et al., 2010	https://software.broadinstitute.org/gatk/
ASCAT	Van Loo et al., 2010	https://www.crick.ac.uk/peter-van-loo/software/ASCAT
Novalign	Novocraft	http://www.novocraft.com
Polysolver	Shukla et al., 2015	http://archive.broadinstitute.org/cancer/cga/polysolver
netMHCpan-2.8	Hoof et al., 2009, Nielsen et al., 2003	http://www.cbs.dtu.dk/services/NetMHCpan-2.8/
netMHC4.0	Andreatta and Nielsen, 2016, Hoof et al., 2009, Nielsen et al., 2003	http://www.cbs.dtu.dk/services/NetMHC/
LOHHLA	This paper	https://bitbucket.org/mcgranahanlab/lohhla

### Contact for Reagent and Resource Sharing

Further information and requests for reagents may be directed to, and will be fulfilled by the Lead Contact, Charles Swanton (charles.swanton@crick.ac.uk).

### Experimental Model and Subject Details

The TRACERx 100 cohort comprises the first 100 patients prospectively analyzed by the lung TRACERx study (https://clinicaltrials.gov/ct2/show/NCT01888601, approved by an independent Research Ethics Committee, 13/LO/1546) and mirrors the prospective 100 patient cohort described in Jamal-Hanjani et al. (2017).

The clinical details of the cohort are described in detail in Jamal-Hanjani et al. (2017). In total, 38 patients were female, while 62 were male. The median age at diagnosis was 68 (range, 34-85).

### Method Details

#### LOHHLA (Loss Of Heterozygosity in Human Leukocyte Antigen) algorithm

As input, LOHHLA requires: a tumor and germline BAM; patient-specific HLA calls, either predicted by an HLA inference tool (e.g., POLYSOLVER [Shukla et al., 2015] or Optitype [Szolek et al., 2014]) or through HLA serotyping; the HLA fasta file location; purity and ploidy estimates. (For implementation of LOHHLA in this manuscript, ASCAT was used to estimate tumor purity and ploidy, while HLA inference was performed using POLYSOLVER, see below.)

To call HLA LOH, LOHHLA relies upon five computational steps:

##### Step 1: extract HLA reads

First, tumor and germline reads that map to the HLA region of the genome (chr6:29909037-29913661, chr6:31321649-31324964, and chr6:31236526-31239869) as well as chromosome 6 contigs (chr6_cox_hap2, chr6_dbb_hap3, chr6_mann_hap4, chr6_mcf_hap5, chr6_qbl_hap6, chr6_ssto_hap7) are extracted using samtools view. Unpaired mates from this step are removed and the output is converted to FASTQ format.

##### Step 2: create HLA allele specific BAM files

For each of the patient’s heterozygous HLA alleles, a patient-specific reference fasta is created. The FASTQ files generated in the previous step are used to generate HLA specific BAM files,using similar mapping parameters to those previously published that allow for reads to map to multiple HLA alleles (Shukla et al., 2015). Post-alignment filtering is subsequently performed such that reads whose mates map to a different allele are discarded, as well as any reads that contain more than one insertion, deletion, or mismatch event compared to the reference HLA allele. For each filtered tumor/germline HLA allele-specific BAM file, coverage is then calculated using samtools mpileup.

##### Step 3: determine coverage at mismatch positions between homologous HLA alleles

For each HLA locus, a local pairwise alignment is performed between the two homologous HLA alleles, using the R Biostrings package. From the pairwise alignment, all of the mismatch positions between the two homologs are extracted. The HLA-specific coverage calculated in Step 2 is then used to determine differences in coverage at each of the mismatch positions. An additional file is also generated containing the coverage at every mismatch position, counting each read only once, as to avoid over-counting reads that span more than one mismatch position.

##### Step 4: obtain HLA specific logR and BAF

LogR across each HLA gene is then obtained by binning the coverage across both homologous alleles at 150 base pair intervals, for both tumor and normal. For each bin, the tumor/normal coverage ratio is multiplied by the multiplication factor, M, corresponding to number of unique mapped reads in the germline, divided by the number of unique mapped reads in the tumor region.

The BAF, corresponding to the coverage of HLA allele 1 divided by the coverage of HLA allele 1 + coverage of HLA allele 2, is subsequently calculated at each polymorphic site.

##### Step 5: determine HLA haplotype specific copy number

Finally, at each polymorphic site, an estimate of the major and minor allele copy number is obtained using the following equations:

$$Allele\;1=\frac{\rho -1+BAF\times 2^{\text{logR}}\times \left(2\left(1-\rho \right)+\rho \times \psi \right)}{\rho }$$

$$Allele\;2=\frac{\rho -1-2{\left(BAF-1\right)}^{\text{logR}}\times \left(2\left(1-\rho \right)+\rho \times \psi \right)}{\rho }$$

where ρ = tumor purity and ψ = tumor ploidy, which are input at the start. The logR value from the corresponding bin in which the polymorphic site was found to reside is used as well as the BAF of the polymorphic site.

For each bin, the median Allele 1 and Allele 2 copy number is then determined. To estimate copy number of Allele 1, the median value across bins is calculated. Likewise, to estimate the copy number of Allele 2, the median value across bins is calculated.

A copy number < 0.5, is classified as subject to loss, and thereby indicative of LOH. To avoid over-calling LOH, we also calculate a p value relating to allelic imbalance for each HLA gene. This p value corresponds to the pairwise difference in logR values at mismatch sites between the two HLA homologs, adjusted to ensure each sequencing read is only counted once. Allelic imbalance is determined if p < 0.01 using the paired Student’s t-Test between the two distributions.

#### TRACERx 100 Cohort

TRACERx samples considered were obtained from (Jamal-Hanjani et al., 2017). Four patients were excluded due to homozygosity at all three HLA loci or too few mismatch positions between HLA alleles. Lung adenocarcinoma and lung squamous cell carcinoma tumors were considered for downstream analyses. Seven tumors were classified as having a separate histology. Of these, one carcinosarcoma exhibited HLA LOH and three adenosquamous carcinomas, one carcinosarcoma, one large cell carcinoma, and one large cell neuroendocrine tumor did not.

#### TRACERx mutation and copy number data

TRACERx mutation data was obtained from Jamal-Hanjani et al. (2017). In brief, mutations were called using VarScan2 (Koboldt et al., 2012) and MuTect (1.1.4) (Cibulskis et al., 2013). To estimate whether mutations were clonal or subclonal, a modified version of PyClone was implemented (Roth et al., 2014). ASCAT (Van Loo et al., 2010) segmented copy number data, purity and ploidy estimates were obtained from Jamal-Hanjani et al. (2017).

To compare LOHHLA to additional tools, we also implemented Sequenza (Favero et al., 2015), and TITAN (Ha et al., 2014). In both cases, default settings were used. For TITAN, the purity estimates from ASCAT were used as input.

#### Comparison of ASCAT and LOHHLA

In order to compare ASCAT and LOHHLA we treated each tumor region as a separate sample, and ran it through the LOHHLA pipeline with default settings. Note, for this analysis we used all TRACERx samples available, including NSCLCs that were not classified as lung adenocarcinomas or lung squamous cell carcinomas.

Given that it was not possible to directly infer the copy number of the HLA alleles using ASCAT, the segment overlapping the HLA locus was used. In twenty-five tumor regions from seven tumors no segment overlapped the HLA locus, and in these cases, the closest genomic segment was used.

To compare our allelic imbalance estimates, we considered a tumor region to be concordant if ASCAT predicted allelic imbalance across the locus and at least one HLA gene using LOHHLA was found to harbor allelic imbalance. Likewise, for LOH, we considered ASCAT and LOHHLA estimates to be concordant if ASCAT predicted a minor allele of 0 and this was also predicted for at least one HLA gene.

Conversely, allelic imbalance estimates were classified as discordant if allelic imbalance was predicted in any HLA gene using LOHHA and not with ASCAT. Similarly, LOH was classified as discordant if any HLA gene using LOHHLA was classified as exhibiting a minor allele of 0 and no LOH was identified using ASCAT.

#### Fragment analysis validation of LOHHLA results

Allelic imbalance was validated using four polymorphic Sequence-Tagged Site (STR) markers located on the short arm of chromosome 6, close to the HLA locus - (D6S2852, D6S2872, D6S248 and D6S1022). 20ng of patient germline and tumor region DNA was amplified using the PCR. The PCR comprised of 35 cycles of denaturing at 95C for 45 s, followed by an annealing temperature of 55C for 45 s and then a PCR extension at 720C for 45 s. PCR products were separated on the ABI 3730xl DNA analyzer. Fragment length and area under the curve of each allele was determined using the Applied Biosystems software GeneMapper v5. When two separate alleles were identified for a particular marker, the fragments could be analyzed for allelic imbalance using the formula (A_tumor_/B_tumor_)/(A_normal_/B_normal_). The output of this formula was defined as the normalized allelic ratio.

#### HLA Type, HLA Mutations, and Predicted NeoAntigen Binders

The HLA type for each sample was inferred using POLYSOLVER (POLYmorphic loci reSOLVER), which uses a normal tissue BAM file as input and employs a Bayesian classifier to determine genotype (Shukla et al., 2015). HLA mutations in each tumor region were also assessed using POLYSOLVER.

Novel 9-11-mer peptides that could arise from identified non-silent mutations present in the sample (Jamal-Hanjani et al., 2017) were determined. The predicted IC_50_ binding affinities and rank percentage scores, representing the rank of the predicted affinity compared to a set of 400,000 random natural peptides, were calculated for all peptides binding to each of the patient’s HLA alleles using netMHCpan-2.8 and netMHC-4.0 (Andreatta and Nielsen, 2016, Hoof et al., 2009, Nielsen et al., 2003). Putative neoantigen binders were those peptides with a predicted binding affinity < 500nM or rank percentage score < 2%.

#### Mapping HLA LOH to phylogenetic trees and identification of parallel evolution

LOH events detected in every tumor region tested were considered to be clonal events and mapped to the trunk of the phylogenetic tree. For heterogeneous LOH events, the regional copy number of the HLA allele lost was used in conjunction with the patient tree structure and subclone cancer cell fractions in a quadratic programming approach, using the R package quadprog, to determine the best placement of the LOH event.

This was achieved by solving a quadratic programming equation:

$$\min\left(-d^{\wedge }T\;b+1/2b^{\wedge }T\;D\;b\right)$$

with the constraints:

$$A^{\wedge }T\;b>=\mathrm{bvec}.$$

The LOH event was tested at each branch. For each possibility, the phylogenetic tree was broken into two, one containing all clones after the LOH event and the other consisting of the remainder of the tree. A 2xn matrix, where n is the number of regions sampled, was constructed containing the regional sum of the cancer cell fractions for each subclone in the subtree and the regional sum of cancer cell fractions from subclones in the remaining tree. The regional cancer cell fraction matrix was multiplied by the transpose of itself to generate a 2x2 matrix for input (*Dmat*) into the quadprog function, solve.QP. The vector to be minimized (*dvec*) was obtained by multiplying the LOHHLA calculated HLA allele copy number for each region by the transpose of the regional cancer cell fraction matrix. Finally, the solve.QP function was called with *Dmat* and *dvec*, using a constraint matrix, *Amat*, such that all results had to be positive and a constraint vector, *bvec*, such that the estimated copy number of HLA allele for the remaining tree was at least 0.5. The errors between observed and predicted copy number values from placing LOH event on each branch were output and the solution providing the least error was selected.

Each mapped event was inspected and events that did not fit the phylogenetic tree or had large error values, either indicating the presence of an additional subclone or multiple independent HLA LOH events, were manually adjusted. Patients CRUK0013, CRUK0061, CRUK0082, and CRUK0084 had HLA LOH events that did not fit the current phylogentic tree, so additional nodes (indicated in gray) were included to contain the HLA LOH event. Patients CRUK003, CRUK0032, CRUK0051, and CRUK0062 had multiple independent HLA LOH events which were manually mapped.

#### Assessing significance of focal and arm-level LOH

In order to assess whether HLA LOH occurred more than expected by chance, we considered whether each LOH event was focal or arm-level in nature. In brief, to classify LOH as arm-level or focal, we focused on the minor allele frequency across the genome. First, any segments (as predicted by ASCAT) with identical minor allele copy numbers were merged. Subsequently, segments that spanned > = 75% the length of a given chromosome arm, were classified as ‘arm-level’, while segments that were < 75% were considered focal.

To assess the significance of focal events, for each tumor, the proportion of the genome subject to focal minor allele loss was determined. This value was assumed to reflect the probability for focal minor allele loss in each tumor. Based on this probability, we generated an aberration state (loss or no loss) for each sample separately and determined the proportion of samples exhibiting loss. We repeated this process 10,000 times to obtain a background distribution reflecting the likelihood of observing losses given the probability of loss in each sample. A p value reflecting the likelihood of observing the level of minor allele loss seen at the HLA locus was determined by counting the percentage of simulations showing a higher proportion loss than that observed.

The same procedure was conducted for arm-level events, using the observed frequency of arm-level allele specific loss in each tumor.

#### Mutational signature analysis

Mutational signatures were estimated using the deconstructSigs R package (Rosenthal et al., 2016). Signature 1A, 2, 4, 5, 13 were considered.

#### Assessing whether neoantigens preferentially bind to loss HLA alleles

To assess whether neoantigens preferentially bind to lost HLA alleles, we focused on tumors exhibiting six distinct HLA alleles (i.e., no homozygosity for any allele in the germline) and loss of one HLA haplotype (HLA-A, HLA-B and HLA-C) in at least one tumor region.

Neoantigens (as defined above), were ranked according to IC_50_ binding scores. Duplicate mutations were removed to ensure each neoantigen reflected the highest binding score (lowest IC_50_ value) for any given mutation. We further filtered the mutation list to only include subclonal mutations (defined as previously described (Jamal-Hanjani et al., 2017)) occurring in the tumor regions harboring loss events (> 5% VAF). The number of subclonal neoantigens binding to each haplotype was then determined for each tumor. A paired wilcoxon test was used to compare the number of subclonal neoantigens binding to the lost haplotype compared to the kept haplotype.

#### PD-L1 immunohistochemistry

Formalin-fixed, paraffin-embedded (FFPE) tissue sections of 4-um thickness were stained for PD-L1 with an anti-human PD-L1 rabbit monoclonal antibody (clone SP142; Ventana, Tucson, AZ) on an automated staining platform (Benchmark; Ventana) with the OptiView DAB IHC Detection Kit and the OptiView Amplification Kit (Ventana Medical Systems Inc.) in a GCP-compliant central laboratory (Targos Molecular Pathology GmbH). PD-L1 expression was evaluated on tumor cells and tumor-infiltrating immune cells. For tumor cells the proportion of PD-L1-positive cells was estimated as the percentage of total tumor cells. For tumor-infiltrating immune cells, the percentage of PD-L1-positive tumor-infiltrating immune cells occupying the tumor was recorded. Scoring was performed by a trained histopathologist [according to previously published scoring criteria (Herbst et al., 2014)].

#### Analysis of TCGA mutation data

TCGA tumor and matched germline exome sequencing BAM files for both lung adenocarcinoma (LUAD, n = 397) and lung squamous cell carcinoma (LUSC, n = 350), were obtained from the Cancer Genome Atlas (TCGA, https://cancergenome.nih.gov/) via https://cghub.ucsc.edu. The data was processed as previously described (Jamal-Hanjani et al., 2017).

#### RNA-seq expression analysis using TCGA

RNA-sequencing data was downloaded from the TCGA data portal. For each LUAD and LUSC sample, all available ‘Level_3′ gene-level data was obtained. Previously defined measures of immune infiltration and activity were used to compare the immune microenvironment between tumors exhibiting HLA LOH at all HLA loci and those without any evidence for HLA LOH (Davoli et al., 2017, Li et al., 2016, Rooney et al., 2015). Additionally the expression level of PD-L1, CTLA4, and an IFN score were compared (Herbst et al., 2014, Piha-Paul et al., 2016, Ribas et al., 2015, Tumeh et al., 2014). Significance was determined using a Wilcoxon test and FDR correction. To determine the degree of change between the HLA LOH groups, a ratio of the medians was calculated. For differential expression analysis, the raw RNA-seq read counts were used as input into the R package DESeq2 for analysis. An FDR cutoff of 0.05 was used to determine genes significantly differentially expressed.

### Quantification and Statistical Analysis

All analysis was performed in the R statistical environment version > = 3.2.1. All statistical tests were two-sided and statistical significance was determined if p value was less than 0.05, unless otherwise stated. Comparisons were made using the Fisher’s exact test Figure 2B, as described above for Figure 3, unpaired Wilcoxon test for Figures 4A–4C, and paired Wilcoxon test for Figures 4D and 4E.

### Data and Software Availability

Code to run LOHHLA is available at https://bitbucket.org/mcgranahanlab/lohhla.

## Consortium

TRACERx Consortium: Charles Swanton, Mariam Jamal-Hanjani, Selvaraju Veeriah, Seema Shafi, Justyna Czyzewska-Khan, Diana Johnson, Joanne Laycock, Leticia Bosshard-Carter, Rachel Rosenthal, Pat Gorman, Robert E. Hynds, Gareth Wilson, Nicolai J. Birkbak, Thomas B.K. Watkins, Nicholas McGranahan, Stuart Horswell, Richard Mitter, Mickael Escudero, Aengus Stewart, Peter Van Loo, Andrew Rowan, Hang Xu, Samra Turajlic, Crispin Hiley, Christopher Abbosh, Jacki Goldman, Richard Kevin Stone, Tamara Denner, Nik Matthews, Greg Elgar, Sophia Ward, Marta Costa, Sharmin Begum, Ben Phillimore, Tim Chambers, Emma Nye, Sofia Graca, Maise Al Bakir, Kroopa Joshi, Andrew Furness, Assma Ben Aissa, Yien Ning Sophia Wong, Andy Georgiou, Sergio Quezada, John A. Hartley, Helen L. Lowe, Javier Herrero, David Lawrence, Martin Hayward, Nikolaos Panagiotopoulos, Shyam Kolvekar, Mary Falzon, Elaine Borg, Teresa Marafioti, Celia Simeon, Gemma Hector, Amy Smith, Marie Aranda, Marco Novelli, Dahmane Oukrif, Sam M. Janes, Ricky Thakrar, Martin Forster, Tanya Ahmad, Siow Ming Lee, Dionysis Papadatos-Pastos, Dawn Carnell, Ruheena Mendes, Jeremy George, Neal Navani, Asia Ahmed, Magali Taylor, Junaid Choudhary, Yvonne Summers, Raffaele Califano, Paul Taylor, Rajesh Shah, Piotr Krysiak, Kendadai Rammohan, Eustace Fontaine, Richard Booton, Matthew Evison, Phil Crosbie, Stuart Moss, Faiza Idries, Leena Joseph, Paul Bishop, Anshuman Chaturved, Anne Marie Quinn, Helen Doran, Angela Leek, Phil Harrison, Katrina Moore, Rachael Waddington, Juliette Novasio, Fiona Blackhall, Jane Rogan, Elaine Smith, Caroline Dive, Jonathan Tugwood, Ged Brady, Dominic G. Rothwell, Francesca Chemi, Jackie Pierce, Sakshi Gulati, Babu Naidu, Gerald Langman, Simon Trotter, Mary Bellamy, Hollie Bancroft, Amy Kerr, Salma Kadiri, Joanne Webb, Gary Middleton, Madava Djearaman, Dean Fennell, Jacqui A. Shaw, John Le Quesne, David Moore, Apostolos Nakas, Sridhar Rathinam, William Monteiro, Hilary Marshall, Louise Nelson, Jonathan Bennett, Joan Riley, Lindsay Primrose, Luke Martinson, Girija Anand, Sajid Khan, Anita Amadi, Marianne Nicolson, Keith Kerr, Shirley Palmer, Hardy Remmen, Joy Miller, Keith Buchan, Mahendran Chetty, Lesley Gomersall, Jason Lester, Alison Edwards, Fiona Morgan, Haydn Adams, Helen Davies, Malgorzata Kornaszewska, Richard Attanoos, Sara Lock, Azmina Verjee, Mairead MacKenzie, Maggie Wilcox, Harriet Bell, Allan Hackshaw, Yenting Ngai, Sean Smith, Nicole Gower, Christian Ottensmeier, Serena Chee, Benjamin Johnson, Aiman Alzetani, Emily Shaw, Eric Lim, Paulo De Sousa, Monica Tavares Barbosa, Alex Bowman, Simon Jordan, Alexandra Rice, Hilgardt Raubenheimer, Chiara Proli, Maria Elena Cufari, John Carlo Ronquillo, Angela Kwayie, Harshil Bhayani, Morag Hamilton, Yusura Bakar, Natalie Mensah, Lyn Ambrose, Anand Devaraj, Silviu Buderi, Jonathan Finch, Leire Azcarate, Hema Chavan, Sophie Green, Hillaria Mashinga, Andrew G. Nicholson, Kelvin Lau, Michael Sheaff, Peter Schmid, John Conibear, Veni Ezhil, Babikir Ismail, Melanie Irvin-sellers, Vineet Prakash, Peter Russell, Teresa Light, Tracey Horey, Sarah Danson, Jonathan Bury, John Edwards, Jennifer Hill, Sue Matthews, Yota Kitsanta, Kim Suvarna, Patricia Fisher, Allah Dino Keerio, Michael Shackcloth, John Gosney, Pieter Postmus, Sarah Feeney, Julius Asante-Siaw, Hugo J.W.L. Aerts, Stefan Dentro, and Christophe Dessimoz

## Author Contributions

N.M. jointly conceived the project, wrote LOHHLA code to perform allele-specific copy number, conducted bioinformatics analysis, supervised the study, and wrote the manuscript. R.R. wrote LOHHLA code, conducted bioinformatics analysis, and wrote the manuscript. C.H. generated the PD-L1 immunohistochemistry data. A.J.R. performed fragment analysis to validate LOHHLA. T.B.K.W., G.A.W., and N.J.B. helped with data analysis. S.V. performed DNA extraction. P.V.L. provided expertise in copy-number analysis. J.H. provided data analysis support and supervision. C.S. jointly conceived the project, supervised the study, and wrote the manuscript with N.M. and R.R. All co-authors contributed to manuscript preparation and research progress discussion.

## Supporting Citations

The following references appear in Figure 6: Spranger et al., 2013, Zaretsky et al., 2016, Zhao et al., 2016.

## Supplemental Information

Table S1. Immune Genes Significantly Differentially Expressed in Lung Adenocarcinomas with HLA LOH versus without HLA LOH, Related to Figure 5

## References

[bib1] Alexandrov L.B., Nik-Zainal S., Wedge D.C., Aparicio S.A., Behjati S., Biankin A.V., Bignell G.R., Bolli N., Borg A., Børresen-Dale A.L., Australian Pancreatic Cancer Genome Initiative, ICGC Breast Cancer Consortium, ICGC MMML-Seq Consortium, ICGC PedBrain (2013). Signatures of mutational processes in human cancer. Nature.

[bib2] Andreatta M., Nielsen M. (2016). Gapped sequence alignment using artificial neural networks: application to the MHC class I system. Bioinformatics.

[bib3] Brastianos P.K., Carter S.L., Santagata S., Cahill D.P., Taylor-Weiner A., Jones R.T., Van Allen E.M., Lawrence M.S., Horowitz P.M., Cibulskis K. (2015). Genomic characterization of brain metastases reveals branched evolution and potential therapeutic targets. Cancer Discov..

[bib4] Brown S.D., Warren R.L., Gibb E.A., Martin S.D., Spinelli J.J., Nelson B.H., Holt R.A. (2014). Neo-antigens predicted by tumor genome meta-analysis correlate with increased patient survival. Genome Res..

[bib5] Campbell J.D., Alexandrov A., Kim J., Wala J., Berger A.H., Pedamallu C.S., Shukla S.A., Guo G., Brooks A.N., Murray B.A., Cancer Genome Atlas Research Network (2016). Distinct patterns of somatic genome alterations in lung adenocarcinomas and squamous cell carcinomas. Nat. Genet..

[bib6] Campoli M., Ferrone S. (2008). HLA antigen changes in malignant cells: epigenetic mechanisms and biologic significance. Oncogene.

[bib7] Carter S.L., Cibulskis K., Helman E., McKenna A., Shen H., Zack T., Laird P.W., Onofrio R.C., Winckler W., Weir B.A. (2012). Absolute quantification of somatic DNA alterations in human cancer. Nat. Biotechnol..

[bib8] Cibulskis K., Lawrence M.S., Carter S.L., Sivachenko A., Jaffe D., Sougnez C., Gabriel S., Meyerson M., Lander E.S., Getz G. (2013). Sensitive detection of somatic point mutations in impure and heterogeneous cancer samples. Nat. Biotechnol..

[bib9] Davoli T., Uno H., Wooten E.C., Elledge S.J. (2017). Tumor aneuploidy correlates with markers of immune evasion and with reduced response to immunotherapy. Science.

[bib10] del Campo A.B., Kyte J.A., Carretero J., Zinchencko S., Méndez R., González-Aseguinolaza G., Ruiz-Cabello F., Aamdal S., Gaudernack G., Garrido F., Aptsiauri N. (2014). Immune escape of cancer cells with beta2-microglobulin loss over the course of metastatic melanoma. Int. J. Cancer.

[bib11] Favero F., Joshi T., Marquard A.M., Birkbak N.J., Krzystanek M., Li Q., Szallasi Z., Eklund A.C. (2015). Sequenza: allele-specific copy number and mutation profiles from tumor sequencing data. Ann. Oncol..

[bib12] Ha G., Roth A., Khattra J., Ho J., Yap D., Prentice L.M., Melnyk N., McPherson A., Bashashati A., Laks E. (2014). TITAN: inference of copy number architectures in clonal cell populations from tumor whole-genome sequence data. Genome Res..

[bib13] Hanahan D., Weinberg R.A. (2011). Hallmarks of cancer: the next generation. Cell.

[bib14] Herbst R.S., Soria J.C., Kowanetz M., Fine G.D., Hamid O., Gordon M.S., Sosman J.A., McDermott D.F., Powderly J.D., Gettinger S.N. (2014). Predictive correlates of response to the anti-PD-L1 antibody MPDL3280A in cancer patients. Nature.

[bib15] Hicklin D.J., Marincola F.M., Ferrone S. (1999). HLA class I antigen downregulation in human cancers: T-cell immunotherapy revives an old story. Mol. Med. Today.

[bib16] Hiraki A., Fujii N., Murakami T., Kiura K., Aoe K., Yamane H., Masuda K., Maeda T., Sugi K., Darzynkiewicz Z. (2004). High frequency of allele-specific down-regulation of HLA class I expression in lung cancer cell lines. Anticancer Res..

[bib17] Hoof I., Peters B., Sidney J., Pedersen L.E., Sette A., Lund O., Buus S., Nielsen M. (2009). NetMHCpan, a method for MHC class I binding prediction beyond humans. Immunogenetics.

[bib18] Jamal-Hanjani M., Wilson G.A., McGranahan N., Birkbak N.J., Watkins T.B.K., Veeriah S., Shafi S., Johnson D.H., Mitter R., Rosenthal R., TRACERx Consortium (2017). Tracking the Evolution of Non-Small-Cell Lung Cancer. N. Engl. J. Med..

[bib19] Koboldt D.C., Zhang Q., Larson D.E., Shen D., McLellan M.D., Lin L., Miller C.A., Mardis E.R., Ding L., Wilson R.K. (2012). VarScan 2: somatic mutation and copy number alteration discovery in cancer by exome sequencing. Genome Res..

[bib20] Lawrence M.S., Stojanov P., Mermel C.H., Robinson J.T., Garraway L.A., Golub T.R., Meyerson M., Gabriel S.B., Lander E.S., Getz G. (2014). Discovery and saturation analysis of cancer genes across 21 tumour types. Nature.

[bib21] Li H., Durbin R. (2009). Fast and accurate short read alignment with Burrows-Wheeler transform. Bioinformatics.

[bib22] Li B., Severson E., Pignon J.C., Zhao H., Li T., Novak J., Jiang P., Shen H., Aster J.C., Rodig S. (2016). Comprehensive analyses of tumor immunity: implications for cancer immunotherapy. Genome Biol..

[bib23] Martin M.P., Carrington M. (2013). Immunogenetics of HIV disease. Immunol. Rev..

[bib24] McGranahan N., Furness A.J., Rosenthal R., Ramskov S., Lyngaa R., Saini S.K., Jamal-Hanjani M., Wilson G.A., Birkbak N.J., Hiley C.T. (2016). Clonal neoantigens elicit T cell immunoreactivity and sensitivity to immune checkpoint blockade. Science.

[bib25] McKenna A., Hanna M., Banks E., Sivachenko A., Cibulskis K., Kernytsky A., Garimella K., Altshuler D., Gabriel S., Daly M., DePristo M.A. (2010). The Genome Analysis Toolkit: a MapReduce framework for analyzing next-generation DNA sequencing data. Genome Res..

[bib26] Mehta A.M., Jordanova E.S., Kenter G.G., Ferrone S., Fleuren G.J. (2008). Association of antigen processing machinery and HLA class I defects with clinicopathological outcome in cervical carcinoma. Cancer Immunol. Immunother..

[bib27] Moretta L., Montaldo E., Vacca P., Del Zotto G., Moretta F., Merli P., Locatelli F., Mingari M.C. (2014). Human natural killer cells: origin, receptors, function, and clinical applications. Int. Arch. Allergy Immunol..

[bib28] Nielsen M., Lundegaard C., Worning P., Lauemøller S.L., Lamberth K., Buus S., Brunak S., Lund O. (2003). Reliable prediction of T-cell epitopes using neural networks with novel sequence representations. Protein Sci..

[bib29] Ott P.A., Hu Z., Keskin D.B., Shukla S.A., Sun J., Bozym D.J., Zhang W., Luoma A., Giobbie-Hurder A., Peter L. (2017). An immunogenic personal neoantigen vaccine for patients with melanoma. Nature.

[bib30] Piha-Paul S.A., Bennouna J., Albright A., Nebozhyn M., McClanahan T., Ayers M., Lunceford J.K., Ott P.A. (2016). T-cell inflamed phenotype gene expression signatures to predict clinical benefit from pembrolizumab across multiple tumor types. J. Clin. Oncol..

[bib31] Ribas A., Robert C., Hodi F.S., Wolchok J.D., Joshua A.M., Hwu W.-J., Weber J.S., Zarour H.M., Kefford R., Loboda A. (2015). Association of response to programmed death receptor 1 (PD-1) blockade with pembrolizumab (MK-3475) with an interferon-inflammatory immune gene signature. J. Clin. Oncol..

[bib32] Rimmer A., Phan H., Mathieson I., Iqbal Z., Twigg S.R.F., Wilkie A.O.M., McVean G., Lunter G., Lunter G., WGS500 Consortium (2014). Integrating mapping-, assembly- and haplotype-based approaches for calling variants in clinical sequencing applications. Nat. Genet..

[bib33] Rizvi N.A., Hellmann M.D., Snyder A., Kvistborg P., Makarov V., Havel J.J., Lee W., Yuan J., Wong P., Ho T.S. (2015). Cancer immunology. Mutational landscape determines sensitivity to PD-1 blockade in non-small cell lung cancer. Science.

[bib34] Rooney M.S., Shukla S.A., Wu C.J., Getz G., Hacohen N. (2015). Molecular and genetic properties of tumors associated with local immune cytolytic activity. Cell.

[bib35] Rosenthal R., McGranahan N., Herrero J., Taylor B.S., Swanton C. (2016). DeconstructSigs: delineating mutational processes in single tumors distinguishes DNA repair deficiencies and patterns of carcinoma evolution. Genome Biol..

[bib36] Roth A., Khattra J., Yap D., Wan A., Laks E., Biele J., Ha G., Aparicio S., Bouchard-Côté A., Shah S.P. (2014). PyClone: statistical inference of clonal population structure in cancer. Nat. Methods.

[bib37] Sahin U., Derhovanessian E., Miller M., Kloke B.P., Simon P., Löwer M., Bukur V., Tadmor A.D., Luxemburger U., Schrörs B. (2017). Personalized RNA mutanome vaccines mobilize poly-specific therapeutic immunity against cancer. Nature.

[bib38] Schumacher T.N., Schreiber R.D. (2015). Neoantigens in cancer immunotherapy. Science.

[bib39] Shen R., Seshan V.E. (2016). FACETS: allele-specific copy number and clonal heterogeneity analysis tool for high-throughput DNA sequencing. Nucleic Acids Res..

[bib40] Shukla S.A., Rooney M.S., Rajasagi M., Tiao G., Dixon P.M., Lawrence M.S., Stevens J., Lane W.J., Dellagatta J.L., Steelman S. (2015). Comprehensive analysis of cancer-associated somatic mutations in class I HLA genes. Nat. Biotechnol..

[bib41] Snyder A., Makarov V., Merghoub T., Yuan J., Zaretsky J.M., Desrichard A., Walsh L.A., Postow M.A., Wong P., Ho T.S. (2014). Genetic basis for clinical response to CTLA-4 blockade in melanoma. N. Engl. J. Med..

[bib48] Spranger S., Spaapen R.M., Zha Y., Williams J., Meng Y., Ha T.T., Gajewski T.F. (2013). Up-regulation of PD-L1, IDO, and T(regs) in the melanoma tumor microenvironment is driven by CD8(+) T cells. Sci Transl Med.

[bib42] Szolek A., Schubert B., Mohr C., Sturm M., Feldhahn M., Kohlbacher O. (2014). OptiType: precision HLA typing from next-generation sequencing data. Bioinformatics.

[bib43] Tran E., Robbins P.F., Lu Y.C., Prickett T.D., Gartner J.J., Jia L., Pasetto A., Zheng Z., Ray S., Groh E.M. (2016). T-cell transfer therapy targeting mutant KRAS in cancer. N. Engl. J. Med..

[bib44] Tumeh P.C., Harview C.L., Yearley J.H., Shintaku I.P., Taylor E.J., Robert L., Chmielowski B., Spasic M., Henry G., Ciobanu V. (2014). PD-1 blockade induces responses by inhibiting adaptive immune resistance. Nature.

[bib45] Van Allen E.M., Miao D., Schilling B., Shukla S.A., Blank C., Zimmer L., Sucker A., Hillen U., Foppen M.H., Goldinger S.M. (2015). Genomic correlates of response to CTLA-4 blockade in metastatic melanoma. Science.

[bib46] Van Loo P., Nordgard S.H., Lingjærde O.C., Russnes H.G., Rye I.H., Sun W., Weigman V.J., Marynen P., Zetterberg A., Naume B. (2010). Allele-specific copy number analysis of tumors. Proc. Natl. Acad. Sci. USA.

[bib47] Yoshihama S., Roszik J., Downs I., Meissner T.B., Vijayan S., Chapuy B., Sidiq T., Shipp M.A., Lizee G.A., Kobayashi K.S. (2016). NLRC5/MHC class I transactivator is a target for immune evasion in cancer. Proc. Natl. Acad. Sci. USA.

[bib49] Zaretsky J.M., Garcia-Diaz A., Shin D.S., Escuin-Ordinas H., Hugo W., Hu-Lieskovan S., Torrejon D.Y., Abril-Rodriguez G., Sandoval S., Barthly L. (2016). Mutations Associated with Acquired Resistance to PD-1 Blockade in Melanoma. N. Engl J. Med.

[bib50] Zhao F., Sucker A., Horn S., Heeke C., Bielefeld N., Schrors B., Bicker A., Lindemann M., Roesch A., Gaudernack G. (2016). Melanoma Lesions Independently Acquire T-cell Resistance during Metastatic Latency. Cancer Res..

